# Quantifying perinatal transmission of Hepatitis B viral quasispecies by tag linkage deep sequencing

**DOI:** 10.1038/s41598-017-10591-9

**Published:** 2017-08-31

**Authors:** Yushen Du, Xiumei Chi, Chong Wang, Jing Jiang, Fei Kong, Hongqing Yan, Xiaomei Wang, Jie Li, Nicholas C. Wu, Lei Dai, Tian-Hao Zhang, Sara Shu, Jian Zhou, Janice M. Yoshizawa, Xinmin Li, Debika Bhattacharya, Ting-ting Wu, Junqi Niu, Ren Sun

**Affiliations:** 1 Department of Molecular and Medical Pharmacology, University of California, Los Angeles, CA 90095 USA; 2 Cancer Institute, Collaborative Innovation Center for Diagnosis and Treatment of Infectious Diseases, School of Medicine, Zhejiang University, Hangzhou, 310058 China; 3 Hepatology, The 1st hospital of Jilin University, Changchun, 130031 China; 4 Key laboratory of Zoonosis Research, Ministry Education, Jilin University, Changchun, 130062 China; 5 Key Laboratory of Infectious Disease, Laboratory of Molecular Virology, Changchun, 130021 China; 6 Epidemiology, The 1st hospital of Jilin University, Changchun, 130031 China; 7 Department of Microbiology and Infectious Disease Center, School of Basic Medical Sciences, Peking University Health Science Center, Beijing, 100191 China; 8 Department of Integrative Structural and Computational Biology, The Scripps Research Institute, La Jolla, CA 92037 USA; 9 Department of Ecology and Evolutionary Biology, University of California, Los Angeles, CA 90095 USA; 10 Molecular Biology Institute, University of California, Los Angeles, CA 90095 USA; 11 Department of Pathology and Laboratory Medicine, David Geffen School of Medicine, University of California, Los Angeles, CA 90095 USA; 12 Department of Medicine, Division of Infectious Diseases, David Geffen School of Medicine, University of California, Los Angeles, CA 90095 USA

## Abstract

Despite full immunoprophylaxis, mother-to-child transmission (MTCT) of Hepatitis B Virus still occurs in approximately 2–5% of HBsAg positive mothers. Little is known about the bottleneck of HBV transmission and the evolution of viral quasispecies in the context of MTCT. Here we adopted a newly developed tag linkage deep sequencing method and analyzed the quasispecies of four MTCT pairs that broke through immunoprophylaxis. By assigning unique tags to individual viral sequences, we accurately reconstructed HBV haplotypes in a region of 836 bp, which contains the major immune epitopes and drug resistance mutations. The detection limit of minor viral haplotypes reached 0.1% for individual patient sample. Dominance of “a determinant” polymorphisms were observed in two children, which pre-existed as minor quasispecies in maternal samples. In all four pairs of MTCT samples, we consistently observed a significant overlap of viral haplotypes shared between mother and child. We also demonstrate that the data can be potentially useful to estimate the bottleneck effect during HBV MTCT, which provides information to optimize treatment for reducing the frequency of MTCT.

## Introduction

Mother-to-child transmission (MTCT) of Hepatitis B Virus (HBV) poses a major obstacle in the mission to eliminate HBV. Despite full immunoprophylaxis (Hepatitis B immune globulin and vaccine series), perinatal transmission still occurs in 2–5% of HBV pregnancies^1–5^. Up to 90% of HBV-infected infants become chronically infected. Individuals with chronic HBV are at high risk of cirrhosis, liver failure, and hepatocellular carcinoma^6, 7^. It is thus essential to understand the factors that contribute to HBV MTCT. High HBV viral load and maternal HBeAg-positivity are the important virologic factors associated with immunoprophylaxis failure in children^1, 2, 5, 7–9^. Additionally, antibody escape viruses that harbor mutations in the major antigenic determinant (“a determinant”) region have also been detected in some immunoprophylaxis failures^3, 10, 11^.

HBV replicates with a mutation rate ~10 times higher than other DNA viruses due to a high replication rate with RNA as the intermediate and a low fidelity polymerase^12–15^. The high mutation rate of HBV causes diversification from the transmitted viral species, yielding a large coexisting pool of genetically heterogeneous but closely related viral genotypes within the same patient. The composition of viral quasispecies may be critical for disease progression and clinical prognosis^16–22^. For example, minor mutations in the basal core promoter and pre-core region have been described in the fulminant course of hepatitis B^23^. Pre-existing drug resistance mutations, even as the minority of the population, have been associated with treatment failure or rapid viral rebound after drug cessation^19, 24, 25^. HBV undergoes rapid intra- and inter-host evolution, especially under selection pressures such as immunoprophylaxis and drug treatment^19^. Investigating the dynamics of viral quasispecies evolution is crucial for the quantitative understanding of how HBV acquires drug resistance or develops antibody escape. Inter-host transmission is generally known as a severe bottleneck of viral evolution, which is proposed to play critical roles in the dynamics of the viral population in the infected individuals and populations^26–28^. Growing evidence shows that only one or a few founder viruses initiate infection for HIV and HCV^26, 27^ and then rapid adaptation and genetic evolution occur in each infected person. However, little is known about the bottleneck of HBV transmission and the evolution of HBV viral quasispecies between mother and child.

**Figure 1:**
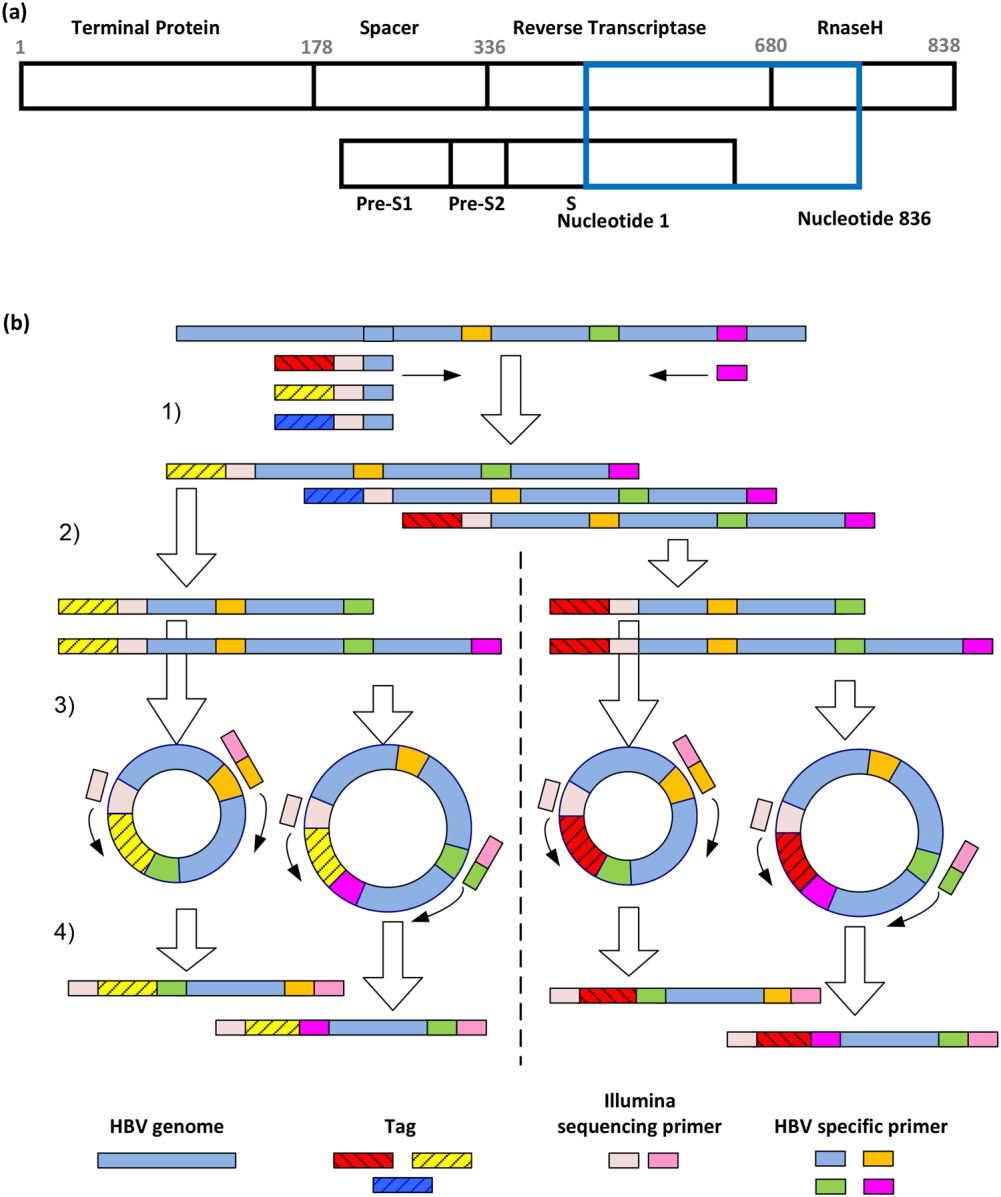
Tag-linkage sequencing of HBV RT/S region. (**a**) A schematic presentation of the HBV viral genome region (polymerase gene) that was processed and reconstructed using the tag-linkage method. 836 bp of polymerase gene (blue box) was recovered, including the majority of the reverse transcriptase domain (overlapping S gene) and part of the RNase H gene. (**b**) A schematic presentation of the experiment flow for tag linkage deep sequencing. 1) HBV sequences were amplified from viral DNA extracted from patient plasma using primers targeting conserved region. Tags were added onto each molecule for sequencing error correction and haplotype reconstruction. 2) Different length of sequences (covering different number of non-overlapping small amplicons) were amplified from ~10,000 molecules. 3) Amplified sequences were ligated. 4) Small amplicons were further amplified for sequencing.

Next-generation sequencing (NGS) provides a rapid and high-throughput method to investigate the diversity of viral populations. However, the short read length and high error rate of NGS poses challenges for the accurate assessment of viral quasispecies of sequences longer than the NGS reads. We recently developed a “tag linkage” method to overcome these two problems^29^. In brief, a 13-nucleotide random tag was assigned to each viral sequence. The long sequences were then divided into small amplicons that contained the same distinct tag, attached by PCR and circulation. The length of each small amplicon could be covered by NGS (Fig. 1). The importance of the randomized tags is two-fold. Firstly, tags can be used for sequencing error correction. The sequencing reads with the same tag are expected to originate from the same template molecule. Thus, the discordance of reads is largely attributed to sequencing error and can be corrected. Secondly, it can be used for haplotype reconstruction. Short reads with the same tag can be clustered to assemble the larger viral genome segments, which enable the determination of viral quasispecies.

In this study, we applied the “tag linkage” method and analyzed the quasispecies of four MTCT pairs that failed immunoprophylaxis. With extensive quality control, the accuracy was validated, which enabled the establishment of the sensitivity of our approach. We reconstructed 836 bp HBV sequences covering the majority of the reverse transcriptase (RT) domain and overlapping S gene with a detection limit of 0.1% of the viral population in each infected individual. Previously identified vaccine escape mutations in the “a determinant” region were observed to be dominant in two children samples. We also observed significant overlap of viral haplotypes between maternal and children samples. Phylogenetic analysis and mathematical simulations suggested that the transmission bottleneck of HBV MTCT is loose, which is in drastic contrast to recent findings of severe transmission bottleneck in HIV and HCV^26, 27^.

## Materials and Methods

### Study subjects and samples

The blood samples were collected from patients at the 1^st^ hospital of Jilin University from July 2012 to February 2015. The study’s protocol was approved by the Research Ethics Committee of the hospital. Informed consents were obtained from patients. This study screened HBsAg positive pregnant females who were followed up by the Department of Hepatology. The infants of HBsAg positive mothers received HBV immunoprophylaxis consisting of Hepatitis B immune globulin (HBIG, 100 IU) and 0.5 ml of the pediatric formulation of HBV vaccine (20 ug, S20133009, Dalian Hissen Bio-pharm. Co., Ltd, Dalian, China). Within two hours after birth, the vaccine and HBIG were given via intramuscular injection. Second and third doses of vaccine were given 1 and 6 months after birth. Blood samples were drawn from the children within 30 to 37 days after the last dose of the vaccine at 7 months ± 1 week of age, and from mothers at delivery. To re-evaluate the positivity of HBsAg, blood samples were drawn from the children again at 12 months of age. HBV serology was done by chemiluminescence technique (Abbott, in the Key Laboratory of Infectious Diseases, Jilin University). HBV viral load was quantified using the COBAS AmpliPrep/COBAS TaqMan assay (Roche Diagnostics, Grenzach, Germany). HBV DNA was extracted from 400ul serum using the EZbead System-32 auto-extraction-system (Haoyuan Biotech, Shanghai, China). In the cohort, 1583 pregnant women were identified as HBsAg positive during the enrollment phase, among which 785 mother-child pairs were successfully followed. 14 children were detected to be HBsAg positive at 7 months ± 1 week of age, and were confirmed to be positive at 12 month of age. Thus, the HBsAg positivity rate of the cohort was 1.8% (14/785). Out of the 14 HBsAg positive children, only 6 had enough blood sample for DNA extraction^30^. All of the corresponding 14 maternal samples had high plasma HBV viral load (>10^7^ IU/ml). 2 out of the 6 children samples failed to be amplified due to low viremia. Thus, the remaining four mother-child pairs (8 samples of the leftovers) were utilized for the current study of the HBV quasispecies transmission. The sequencing and data analyses were done at UCLA with the approval from UCLA IRB. All methods performed were in accordance with the relevant guidelines and regulations.

### Tag-linkage sequencing of HBV RT/S region

For all 8 samples, a 13-bp random nucleotide tag and a unique population ID were linked on the amplified RT/S region through PCR. 500ng extracted DNA were used as a template for amplification. Each sample was then diluted to ~10,000 dsDNA/ul through serial dilution. Approximately 10,000 DNA molecules were re-amplified and separately amplified with different primer sets rendering two small products with length ~450 bp and ~900 bp. Small products were ligated with a 15 bp linker. Ligations were performed in a 100 ul system with 0.1 ng total DNA to maximize self-ligation. Ligation products were then amplified to make the final ~430 bp amplicons. Finally, sequencing adaptors were added onto amplicons for Illumina sequencing. All the primers were designed to be primed at the conserved regions of HBV genome (Supplementary methods).

### Sequencing and Data analysis

Samples were sequenced with 1.5 lanes of Illumina Miseq PE250 (MiSeq Reagent Kit v2, MS-102–2003).

Sequencing data were analyzed with customized python scripts (available upon request). Sequencing reads were first matched to the primer sequence and then separated into different amplicons accordingly. Forward and reverse reads were paired and aligned. For each amplicon, sequences with the same population ID and the same randomized tag were clustered together. Within each cluster, all the sequences were aligned and sequences with different lengths (insertions or deletions) were filtered out. For sequencing error correction, mutations were identified if they occurred in more than 60% of reads within one cluster based on consensus of each patient sample. Haplotypes were then assembled if three or more amplicons occurred for the same tag.

### Statistic and phylogenetic analysis

Shannon entropies were calculated using vegan package in R language. Multiple sequence alignment was performed with Muscle^31^. Phylogenetic trees of each pair of MTCT patients were generated using PhyML under the GTR substitution model. Bootstraps were performed 100 times to examine the confidence of the trees^32, 33^. For better visualization, we drew the branch width as proportional to the bootstrap value, and the thickest branch with a bootstrap value equal to 1. Highlighter plots were generated using the highlighter tool from http://www.hiv.lanl.gov/ ^34^. The numbering of the viral haplotypes (P1M.1, PM1.2, *et al*.) was ranked in accordance to the frequency in the corresponding patient sample, with the number 1 haplotype (example: P1M.1) being the dominant species. All the haplotypes >0.1% were considered for phylogenetic analysis.

### Estimation of the transmission bottleneck

Random multinomial samplings of transmitted viral haplotypes were performed based on the frequency distribution of the mother’s haplotypes^27^. Viral particles were randomly generated with a probability proportional to the relative frequency of each haplotype. The number of haplotypes shared between the mother and the child was defined as the threshold (*N*). For a given number of transmitted infectious particles, we performed 1000 samplings. In each sampling, we counted the number of haplotypes being transmitted. Assuming a given number of transmitted infectious particles, we then calculated the percentage of times (out of 1000 simulations) that the probability of successfully transmitting was equal or more than the number of viral haplotypes shared between mother and child *(N*). To give an estimate for the lower bound of transmission bottleneck, we reported the number of transmitted viral particles that the probability of successfully transmitting the shared haplotypes is larger than 5%. In this calculation, we assume that the diversity of transmitted haplotypes does not impact the efficiency of transmission.

### Nucleotide sequence accession numbers

Raw sequencing reads were deposited at NCBI sequence read archive (SRA) with Bio Project ID: PRJNA309553.

## Results

### Clinical and laboratory information

Four mother-child pairs that failed HBV immunoprophylaxis were available for sequencing analysis, from a cohort that identified 1583 pregnant women as HBV-positive during July 2012 to February 2015 (Method). All 8 individuals were infected with subtype C HBV virus, which is the dominant subtype for Chinese patients. Patient information and HBV statuses are shown in Table 1. All 4 mothers were HBeAg positive, and had high HBV viral load (>10^8^ IU/ml). No antepartum HBV antiviral therapy was received. HBV immunoprophylaxis of children included HBIG (100IU) and 3 doses of pediatric formulation of HBV vaccine (0.5 ml). The first doses of the vaccine and HBIG were administered within two hours after birth. The second and the third dose of HBV vaccine were given at 1 and 6 months post birth, respectively. Blood samples were collected from mothers at delivery, and from the children within 30 to 37 days after the last dose of the vaccine at 7 months ± 1 week of age.

**Table 1:** Patient Information.

	P1M	P1C	P2M	P2C	P3M	P3C	P4M	P4C
HBV DNA (IU/ml)	1.70 × 10^9^	1.06 × 10^8^	3.40 × 10^8^	6.06 × 10^7^	1.03 × 10^8^	1.97 × 10^8^	1.70 × 10^9^	2.64 × 10^8^
HBsAg (IU/ml)	>250	>250	>250	>250	>250	>250	>250	>250
HBsAb (mIU/ml)	0	0.33	0.35	0.06	0	0	0.18	0
HBsAb (positivity)	(−)	(−)	(−)	(−)	(−)	(−)	(−)	(−)
HBeAg (S/CO)	1182.52	324.862	1271.72	244.589	575.309	430.197	1178.93	161.91
HBeAg (positivity)	(+)	(+)	(+)	(+)	(+)	(+)	(+)	(+)
HBeAb (S/CO)	95.21	40.25	97.96	31.6	28.15	22.96	96.43	22.34
HBcAb (S/CO)	6.37	5.55	7.95	4.62	9.17	10.35	5.79	9.4
Dose of Vaccine	/	20ug	/	20ug	/	20ug	/	20ug
Number of Vaccinations	/	3	/	3	/	3	/	3
Age	27 years	7 month ± 1 week	33 years	7 month ± 1 week	25 years	7 month ± 1 week	27 years	7 month ± 1 week

Note: P1M is mother of the first patient pair, P1C is the child first patient pair. And so on.

### Reconstruction of HBV viral RT/S region with tag-linkage method

We analyzed an 836 bp region of the HBV genome covering the majority of polymerase reverse transcriptase domains and the overlapping S gene [Fig. 1A]. This is a diverse region on HBV genome and responded to HBs antibody selection and drug resistance [Supplementary Figure S1]. The “tag-linkage” method, which was recently established and validated, was used in order to get high quality viral haplotypes [Fig. 1B]^29^. Briefly, HBV viral DNAs were extracted and amplified from the sera. Tags containing 13 random nucleotides were then linked to DNA molecules through limited cycles of PCR amplification. The tagged products were then divided into two ~430 bp non-overlapping amplicons, which could be covered by combining forward and reverse reads of Illumina Miseq PE250. The unique tag from the full-length product was linked to each small amplicon through circularization based PCR-amplification. For each patient sample, ~10,000 tagged DNA molecules were processed and deep sequenced. The 13 random nucleotide tag allowed the maximum complexity to be 6.7e^7^ (4^13^), ensuring the separation of ~10,000 molecules through the recognition of tag sequences. All the primers were designed to be primed at the conserved regions of HBV genome (supplementary method). We achieved at least 100,000 reads coverage for each amplicon, which provided ~10 reads per viral sequence for sequencing error correction using the tag. ~3,000–5000 unique tags (30–50% of input tagged DNA molecules) were detected for each amplicon post quality filtering and error correction.

**Figure 2:**
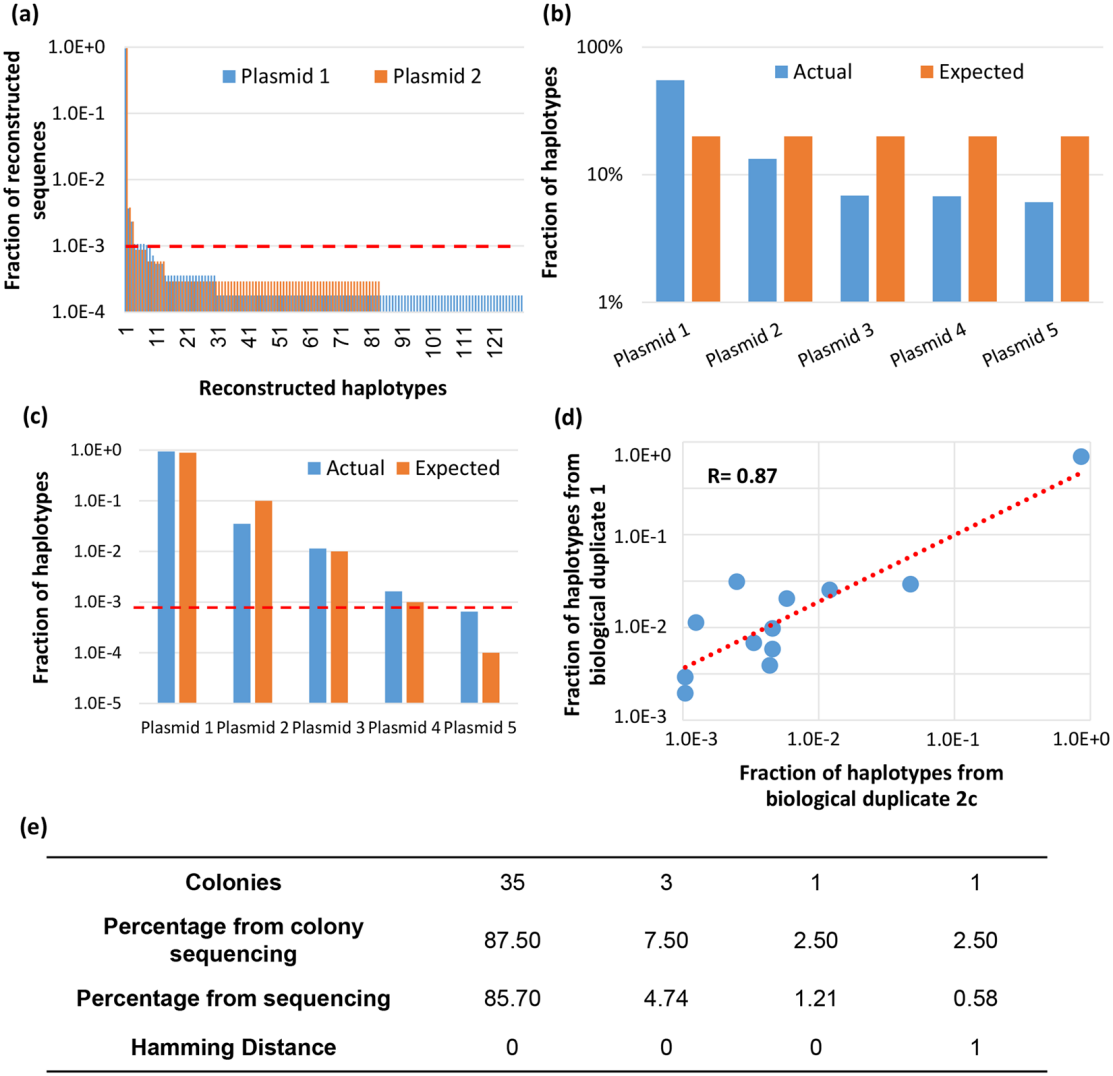
Quality control examination of the tag-linkage method. (**a**) Two HBV clonal plasmids were used to examine processing errors. Fractions of reconstructed sequences are shown. More than 95% of errors result in false positive haplotypes that occur ≤0.1%, thus justifying 0.1% as our detection limit for minor viral haplotypes. (**b**) Successful detection of 5 different haplotypes is shown upon mixture of 5 plasmids in equal ratio. (**c**) Detection of 5 different haplotypes is shown upon mixing 5 plasmids in log ratio. The red dashed line represents the highest frequency of viral haplotypes detected in this sample which does not belong to the parental 5 plasmids, representing the level of noise. (**d**) Correlation of viral haplotypes of biological duplicates of mother sample of patient 1. (**e**) Correlation between clonal sequencing and tag-linkage sequencing. 40 random colonies were selected and their haplotype sequences were analyzed via both methods.

To examine the quality of our “tag-linkage” method, we performed multiple steps of quality control. Firstly, two HBV clonal plasmids were used as input instead of patient DNA to examine the processing errors (PCR and sequencing errors). For both plasmids, ~96% reconstructed full-length sequences were correct. More than 95% of the remaining errors resulted in false positive haplotypes occurring at ≤ 0.1%. Thus, we set 0.1% as our detection limit for a minor viral haplotype [Fig. 2A]. Secondly, the accuracy and sensitivity of our method was examined by mixing 5 different HBV clonal plasmids at equal or different ratios as input. When mixing 5 plasmids in equal ratio (20% of each), we successfully detected all 5 different haplotypes as dominant species [Fig. 2B]. There were some distortions of detected frequency versus actual frequencies, which might have come from PCR bias. We also mixed 5 plasmids with log ratio, with the lowest one at ~0.01%. As expected, haplotypes >0.1% were confidently detected, while the other two at 0.1% and 0.01% were mixed with noise [Fig. 2C]. Thirdly, a biological duplicate of P1M (pair 1 mother) sample from the DNA extraction step was performed. All the viral haplotypes detected >0.1% were repeatedly detected in the biological duplicate, with a ranking correlation coefficient of 0.87 [Fig. 2D]. Lastly, clonal sequencing for P1M sample was conducted to examine the correlation by the tag-linkage sequencing method. We randomly picked 40 colonies and detected 4 different haplotypes. Three of the haplotypes were observed by tag-linkage sequencing method, and the frequency was close to clonal sequencing results [Fig. 2E]. The missing haplotypes were different by one base pair when compared to the closest viral sequence in the quasispecies reconstructed by our approach. The traditional clonal sequencing data verified our tag linkage sequencing method. The above results establish the sensitivity and accuracy of our tag linkage sequencing approach to detect HBV haplotypes.

### Characteristics of Intra-host diversity of HBV viral quasispecies

**Figure 3:**
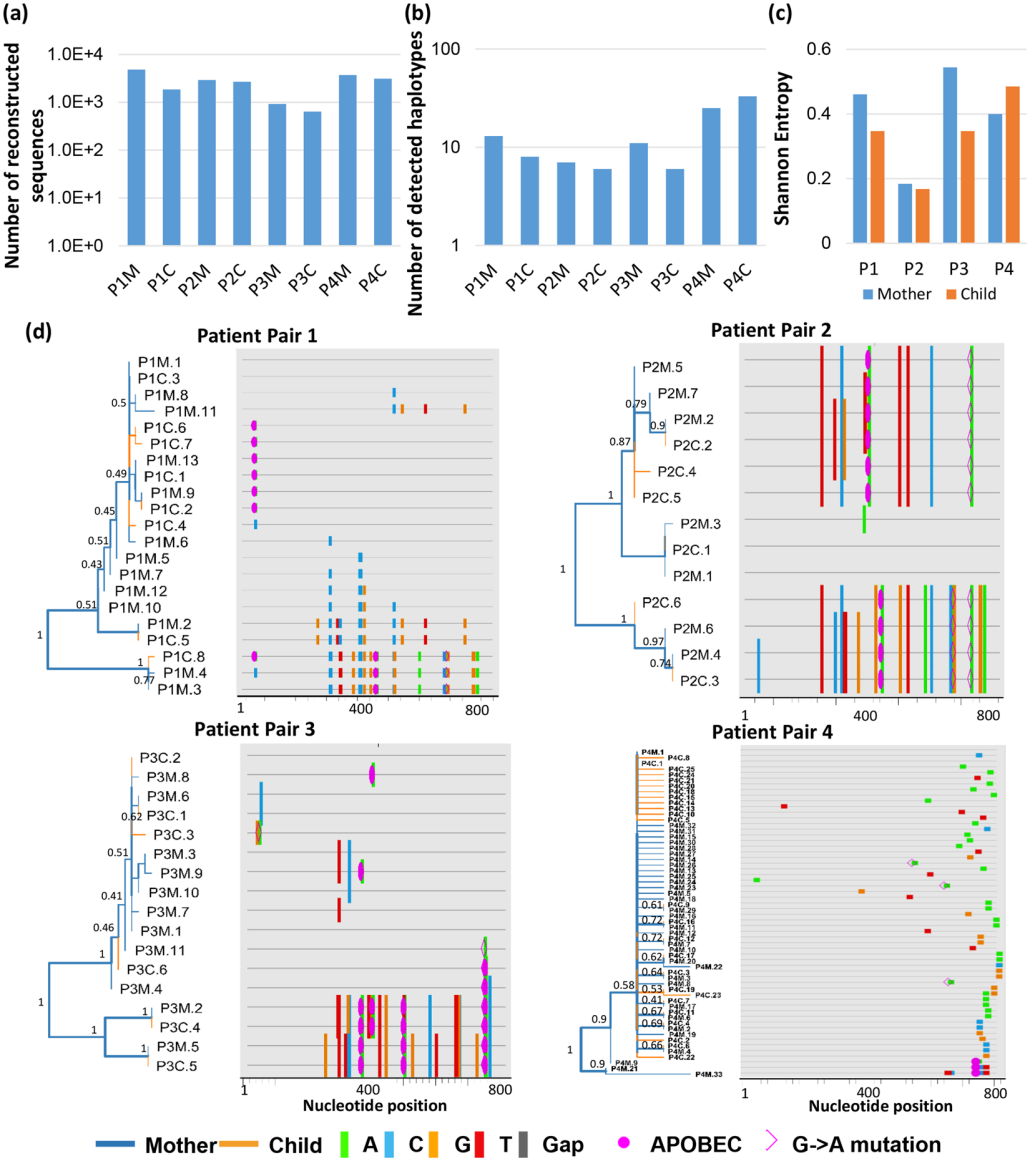
Characteristics of Intra-host diversity of HBV viral quasispecies. (**a**) Numbers of viral sequences that were detected for each sample. P1 stands for the mother-child pair 1, and there are four pairs (P1 to P4). M stands for maternal sample, and C stands for child sample. (**b**) Numbers of high quality viral haplotypes detected in each infected individual. (**c**) Bar plots are shown for the normalized Shannon entropy of viral quasispecies for each patient. (**d**) Phylogenetic tree and highlighter plot are shown for viral haplotypes of each mother-child pair. 100 bootstraps were performed. The tree branch width is proportional to the bootstrap value.

For the 8 samples from four mother-child pairs, the number of viral sequences that were successfully assembled for each sample is shown in Fig. 3A. Based on the results from control plasmids, we focused our analysis on the viral sequences that occurred >0.1%, which were defined as “high quality quasispecies”. Up to 33 different high quality haplotypes were detected within one sample for the sequenced region, which are shown in Fig. 3B.

To examine the diversity of intra-host viral quasispecies, the normalized Shannon entropy (Sn) was calculated for each patient sample. Sn ranged from 0.17–0.55, depending on patients [Fig. 3C]. There was no significant difference of Sn between samples from mother and child (p = 0.57, two-tailed T-test). For each mother-child pair, we constructed the phylogenetic tree by PhyML using GTR substitution model. Bootstraps were performed 100 times to examine the confidence of the trees^32, 33^. The branch width of the tree was proportional to the bootstrap value, with the thickest branch having a bootstrap value equal to 1. Bootstrap values were also labeled for major branches. The numbering of viral haplotype for each patient was ranked by the corresponding frequency. We did not observe any data that suggested a single founder from the maternal samples established the phylogeny of the children’s samples. Instead, the viral haplotypes are mixed and shared between mothers and children for these four patient pairs. Paired with the phylogenetic tree, highlighter plots were also generated to demonstrate the mutations occurring in each haplotype, using the dominant haplotype from maternal sample as the master sequence^34^.

### Genetic drift and transmission bottleneck of HBV quasispecies in MTCT

**Figure 4:**
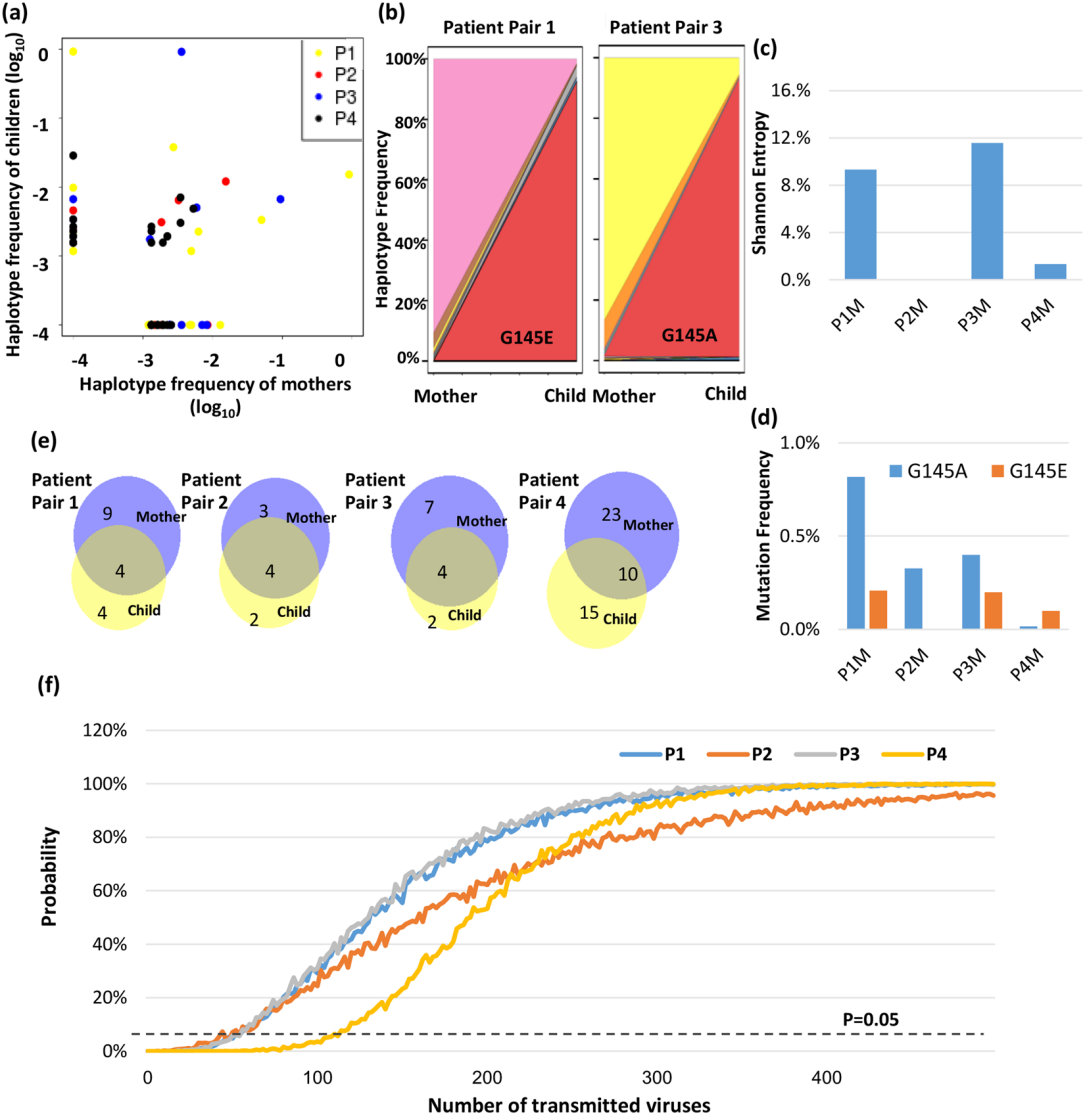
Genetic drift and transmission bottleneck of HBV quasispecies in MTCT. (**a**) The occurrence frequency of each viral haplotype is shown as a scatter plot in the log scale. Haplotypes that were not detected in patients were plotted as −4. (**b**) Relationships of viral haplotypes detected in mother and their child samples for pair 1 and 3. Each color represents one HBV haplotype and their percentage corresponds to its abundance in the viral population in the indicated individual. G145E and G145A were minor quasispecies in mother samples and became dominant in the corresponding child samples. (**c**) Shannon entropy of the “a determinant” region is shown for each maternal sample. (**d**) Frequencies of detected “a determinant” mutations are shown for maternal sample. (**e**) Venn diagrams summarize the overlapping viral haplotypes between maternal and child samples. (**f**) Simulation result depicts the probability of successful transmission of the shared viral haplotypes between mother and child, dependent on the transmission bottleneck. X-axis represents the number of transmitted viruses being sampled in simulations. Y-axis represents the probability of transmitting equal or more than the number of viral haplotypes shared between the mother and the child.

To further investigate the relationships between the viral haplotypes between maternal and child samples, scatter plots were generated for haplotype frequencies for each mother-child pair. We observed that pair 2 and pair 4 shared a significant portion of same haplotypes with similar frequencies, while the major haplotype of mother and child were different for pair 1 and pair 3 [Fig. 4A].

Newly emergent dominant mutations in the child sample for pair 1 and pair 3 were examined for selective sweeping of certain beneficial mutations. Notably, mutations at position G145 harbored the most change. G145A became dominant in the child from pair 3, and G145E for the child from pair 1 [Fig. 4B]. We detected single mutations: G145R, G145K and G145E, as well as double mutations: D144G-G145E and K141R-D144N that occurred more often in children samples. These mutations are located at the 2nd loop of “a determinant” region, and are responsible for escaping from hepatitis B surface antibody recognition^35–39^. The disruption of local structures might change the immunological characteristics of HBsAg and diminish the binding of the antibody, resulting in vaccine escape. Both G145A^12, 40–44^ and G145E^45, 46^ have been detected in HBsAg-positive vaccinated individuals, which are attributed to their antibody escape phenotype. Thus, the distinct proportion of viral quasispecies for pairs 1 and 3 are most likely attributed to the growth advantage of “a determinant” mutations. We further investigated if there was any relationship between the transmission of “a determinant” mutations and the minority variant quasispecies in maternal samples. The entropy of “a determinant” domain was larger for patient 1 and 3, suggesting that these two maternal samples have higher diversity at the antibody recognition region [Fig. 4C]. G145A and G145E were detected in maternal samples with a slightly higher percentage in patients 1 and 3. However, all of them were minor mutations that occurred at a frequency of <1% [Fig. 4D].

Despite the dominance of “a determinant” region mutations for pair 1 and 3, we observed that the overall viral haplotypes were significantly overlapped between mother and child of each pair. For all pairs, close to or more than 50% of viral haplotypes in the maternal samples were also observed in the child samples [Fig. 4E]. Although there are limitations with our current samples and size of the cohort (discussion), with relatively accurate measurement of viral quansispecies, it might be informative for assessing the transmission bottleneck of HBV MTCT. Growing evidence shows that only one or a few viral particles initiate infection during HIV and inter-host transmission during HCV^26, 27^. Supporting evidence comes from the low viral sequence diversity detected immediately after transmission. However, this issue has not been well defined for HBV. To estimate the HBV transmission bottleneck, we applied random multinomial samplings to simulate the process of MTCT (methods)^3, 27, 28^. It is estimated that 50~200 infectious HBV viruses need to be transmitted so that the probability of successfully transmitting the shared haplotypes is larger than 5% [Fig. 4F]. This result indicates that the MTCT bottleneck for HBV might be fairly loose for the four MTCT cases studied her. However, due to the limited number of patient samples available, the late time point that children samples were collected and non-whole genome sequencing of current study, further investigation with a higher quality of whole genome sequencing data is needed to further quantify the genetic bottleneck of MTCT in HBV.

## Discussion

Over 240 million people are chronically infected with HBV, and ~1 million deaths per year are attributed to HBV related diseases^6^. Mother to child transmission of HBV virus is a leading cause of chronic infection. HBV immunoprophylaxis has greatly reduced the MTCT rate from ~50% to 2–5%^2, 40^. However, due to the large number of HBV infected people, the 2–5% immunoprophylaxis failure remains an important public health issue. For the current cohort, 1583 pregnant women were identified as HBsAg-positive and 785 mother-child pairs were followed up. 14 children were detected to be HBV infected. We analyzed the HBV quasispecies evolution in the 4 mother-child transmission pairs that had enough serum for successful PCR amplification. The tag-linkage method enabled us to accurately measure mutations in minor subpopulations and assemble long viral haplotypes. We observed that “a determinant” mutations were dominant in two children samples that pre-existed as minor quasispecies in corresponding maternal samples. Besides the selection sweep of “a determinant” mutations, limited evolution or genetic drift was observed in the viral population from mother to child. Phylogenetic analysis and mathematical simulation estimated that 50~200 infectious HBV viruses need to be transmitted, suggesting a loose bottleneck during HBV MTCT.

Mutations in HBV surface protein are known to play an important role in antibody escape and vaccine failure. Accurate identification of minor mutations is critical for determining the relationship between pre-existence of mutations in maternal samples with the dominance of mutations in child samples. Taking advantage of the tag-linkage method, we were able to detect minor haplotypes at a frequency of ~0.1% by correcting sequencing errors. We observed a slightly higher diversity at “a determinant” regions and a higher frequency of possible vaccine escape mutations in mothers who transmitted mutated strains to their children.

Our data and simulations suggest that 50~200 viruses need to be transmitted to generate the observed quasispecies pattern. This contrasts with HIV and HCV, in which only one or a few founder viruses can get through the severe MTCT bottleneck and establish infection. However, it is similar with the estimation for influenza transmission^26–28^. A number of factors might contribute to the loose bottleneck with a transmission of multiple founder viruses. Firstly, all 4 mothers in our study were HBeAg positive, with HBV copy number >10^8^ /ml. Rapid viral replication increases the risk of intrauterine transmission through the induction of T cell tolerance in utero^2^. Moreover, the high viral load of the mother may expose the newborn to a large quantity of viruses that exceed the neutralizing ability of antibodies. Furthermore, recent studies suggested HCV and HIV founder viruses are characterized with strong immune escape properties due to the robust selection during transmission^26, 47–50^. However, natural infection of HBV contains a HBeAg-positive immunotolerant phase for mother and child^51^. The lack of immune pressure may cause a lack of selection in the founder virus. Finally, HBV has much higher infectivity (~50–100 times higher than HIV) that enable the transmission of large amount of viruses through blood exchange^52^. The MTCT without immunoprophylaxis for HBV is >90%, compare to 5–7% for HCV and 15–45% for HIV^53–55^.

The loose bottleneck that is observed here might also provide support and guidance for the treatment of HBV during the third trimester. If actual transmission of virus is partly due to the inefficient neutralizing ability of antibodies, then the quantity of transmitted virus might be proportional to maternal viral loads. In this scenario, for patients with HBV copy number >10^8^ /ml, it might be necessary to reduce the viral load more than 50~200 fold to reduce the possibility of MTCT. This hypothesis is also consistent with our cohort data, in which all the MTCT cases occur in the mother sample with HBV copy number >10^7^ /ml.

Decreasing the viral copy number through drug treatment during the third trimester might be efficient in reducing MTCT rate^56–58^. One major concern of drug treatment was the selection of drug resistant mutations in the reverse transcriptase and the corresponding HBs mutations that confer antibody escape. To assess the possibility of drug resistance, we analyzed the frequency of well-known drug (Nucleos(t)ide Analogues) resistant mutations on position rt171, rt173, rt180, rt181, rt204 in maternal samples [Supplementary Figure S2]. They only existed at very low frequencies (<0.2%). Moreover, we did not detect the co-occurrence of drug resistant mutation and antibody escape mutation on one haplotype in any patient samples.

As the first comprehensive examination of the viral quasispecies during MTCT, additional studies are required to address several limitations of this study. Firstly, this study was restricted to subtype C. It is to be determined whether the loose bottleneck will be observed for patients with other subtypes of HBV infection. Secondly, the children samples were collected ~7 months after birth, which may already contain mutations compared with the viral haplotypes immediately after transmission. Based on the reported HBV mutation rate, 1 mutation at most, would occur in the region that was examined during the 7 months^12–15^. Thus, we assume it would not have severe impact on the main conclusion of our analyses. We cannot rule out the possibility of convergent evolution or extinction of viral haplotypes in children, but the substantial overlap of viral haplotypes between mother and child even after 7 months post-birth leads to the observation of a loose transmission bottleneck. The large number of quasispecies deduced from a long segment of the viral genome suggested that these quasispecies were not generated *de novo* in children. The most possible explanation is that they were transmitted from their mothers, still persisted in the infant at month 7 post birth, and evolve to have some diversity in the infants. More children samples collected immediately after birth are most suitable to rarify our observation. Lastly, although there is much improvement in the accuracy and sensitivity of the tag-linkage method on detecting minor viral haplotypes, it has drawbacks with extensive PCR amplification that might create some distortion of the ratio of a subset of viral haplotypes. A transposon-based system or endonuclease system might be incorporated to control the bias during PCR amplification, though it is more technically intensive^59^.

In summary, we comprehensively and quantitatively analyzed the HBV viral quasispecies evolution in mother-child transmission samples with the “tag-linkage” method. Dominance of “a determinant” mutations was observed and a significant overlap of viral haplotypes was shared between mother and child, suggesting the transmission bottleneck during MTCT might be loose. Further studies with more MTCT samples collected shortly after birth and with different serotypes should be studied to verify our observation. Moreover, the “tag-linkage” method presented here is widely applicable to study infections, transmission and evolution of other types of viruses.

## Electronic supplementary material

Supplementary Information
