# Supplementary material for: Quantifying perinatal transmission of Hepatitis B viral quasispecies by tag linkage deep sequencing
Source: Sci Rep. 2017 Aug 31;7:10168. doi: 10.1038/s41598-017-10591-9 (PMC5578979; doi:10.1038/s41598-017-10591-9)
Supplement: Supplementary file 1 — Supplementary Information [file 41598_2017_10591_MOESM1_ESM.pdf]

Quantifying perinatal transmission of Hepatitis B viral quasispecies by tag linkage deep sequencing

Running Title: HBV quasispecies transmission

Yushen Du[1,2§], Xiumei Chi[3,4,5§], Chong Wang[3], Jing Jiang[6], Fei Kong[3], Hongqing Yan[3], Xiaomei Wang[3,4,5], Jie Li[7], Nicholas C. Wu[1,8], Lei Dai[1,9], Tianhao Zhang[1,10], Sara Shu[1], Jian Zhou[11], Janice M. Yoshizawa[11], Xinmin Li[11], Debika Bhattacharya[12], Ting-ting Wu[1], Junqi Niu[3,4,5*], Ren Sun[1,2,9*]

1, Department of Molecular and Medical Pharmacology, University of California, Los Angeles, CA 90095, USA

2, School of Medicine, Zhejiang University, Hangzhou 310058, China

3, Hepatology, The 1st hospital of Jilin University, Changchun 130031, China

4, Key laboratory of Zoonosis Research, Ministry Education, Jilin University, Changchun 130062, China

5, Key Laboratory of Infectious Disease, Laboratory of Molecular Virology, Changchun 130021, China

6, Epidemiology, The 1st hospital of Jilin University, Changchun 130031, China

7, Department of Microbiology and Infectious Disease Center, School of Basic Medical Sciences, Peking University Health Science Center, Beijing 100191, China

8, Department of Integrative Structural and Computational Biology, The Scripps Research Institute, La Jolla, CA 92037, USA

9. Department of Ecology and Evolutionary Biology, University of California, Los Angeles, CA 90095, USA

10, Molecular Biology Institute, University of California, Los Angeles, CA 90095, USA

11, Department of Pathology and Laboratory Medicine, David Geffen School of Medicine, University of California, Los Angeles, CA 90095, USA

12, Department of Medicine, Division of Infectious Diseases, David Geffen School of Medicine, University of California, Los Angeles, CA 90095, USA

§These authors contribute equally to this work

Corresponding authors

Junqi Niu junqiniu@aliyun.com Ren Sun rsun@mednet.ucla.edu

Supplementary Figures

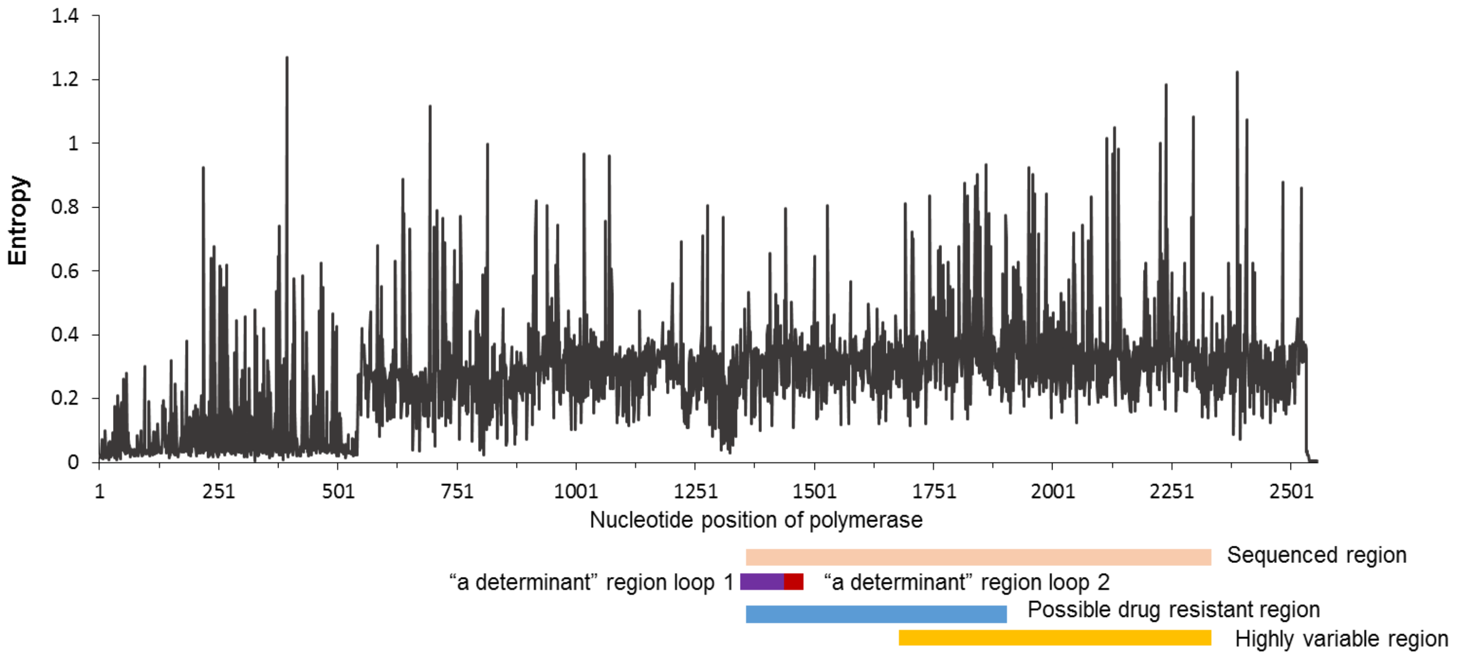

Supplementary Figure S1: Entropy at each nucleotide position of HBV Pol

Entropy of each nucleotide position of HBV Pol are shown for cross-sectional data. Regions being sequenced and analyzed in current study are highlighted.

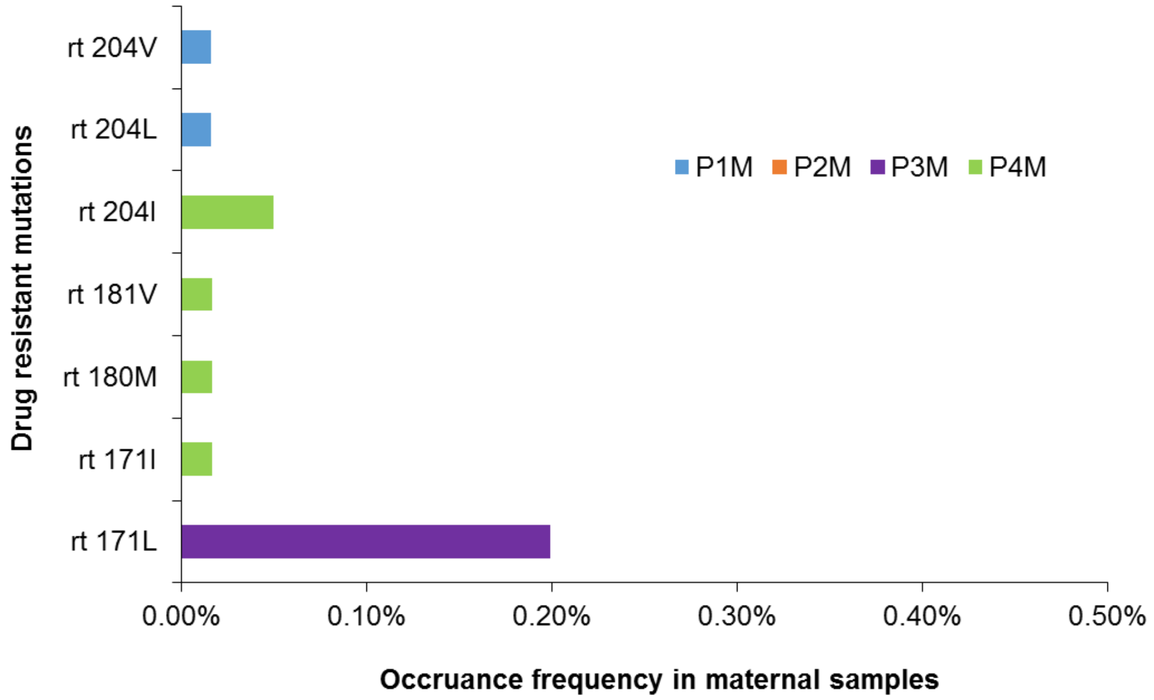

Supplementary Figure S2: Occurrences of drug resistant mutations

Frequencies of well-known drug resistant mutations on position rt171, rt173, rt180, rt181, rt204 in maternal samples.

Supplementary methods

Detailed protocol for tag-linkage sequencing for HBV RT/S region

We used KOD hot start DNA enzyme for all the amplification steps (Millipore Cat 71086)

1) Amplify HBV sequence from extracted DNA sample with the following primer. Underlined are illumina sequencing primer (constant region), bolded are HBV specific annealing region. The numbering of primers are according to Hepatitis B virus DNA complete genome isolate:AK66 (Sequence ID: AB033556.1, subtype C)

HBVRTforward_TAG	AGATCGGAAGAGCGTCGTGTAGGG**AATCTTCTCGAGGACTGGGGACCCTGCACC (120-149)**
HBVrtReverse	**GAG CAG CCA TGG GAA GGA GGT GTA TTT CCG (1353-1382)**

95 ℃ 30 sec, 62℃ 30sec, 68℃ 1min 30sec 25 cycles;

2) Adding randomized tag and population ID on to each sample. The highlighted region is population ID, which is different for each patient. Italicized are SapI digestion sites, underlined are illumina sequencing primer region, similar as the 1st PCR.

HBVrt_TAG_GCA	TAGATACTAT *GCTCTTC* GCAA NNNNNNNNNNNNNN GCA AGATCGGAAGAGGGTCGTGTAGGG
HBVrtReverse	**GAG CAG CCA TGG GAA GGA GGT GTA TTT CCG (1353-1382)**

95 ℃ 30 sec, 62℃ 30sec, 68℃ 1min 30sec 20 cycles

3) Dilute the 2nd PCR product to 10,000 molecular per microliter, and then use 1ul as template for 3rd PCR to re-amplify the tagged viral sequences.

ErrorCorrect_SapI	ACA TAGATACTAT *GCTCTTC* GCAA
HBVrtReverse	**GAG CAG CCA TGG GAA GGA GGT GTA TTT CCG (1353-1382)**

95 ℃ 30 sec, 56℃ 30sec, 68℃ 1min 30sec 25 cycles

4) Amplify the small amplicons with the following primer sets. Italicized are SapI digestion sites

Amplicon	Forward Primer	Reverse Primer
Amplicon 1	ACA TAGATACTAT *GCTCTTC* GCAA	CA *GCTCTTC* AGGT **TTGACAKACTTTCCAATCAAT (972-992)**
Amplicon 2	ACA TAGATACTAT *GCTCTTC* GCAA	CA *GCTCTTC* AGGT **GAGCAGCCATGGGAAGGAGGT (1353-1373)**

Amplicon 1: 95 ℃ 30 sec, 56℃ 30sec, 68℃ 1min 20 cycles

Amplicon 2: 95 ℃ 30 sec, 56℃ 30sec, 68℃ 1min 30sec 20 cycles

5) For each small amplicon, digested with SapI enzyme and ligated with 15bp linker.

Linker is generated by annealing the following two oligos:

Linker_ F /5phos/TTG CCGACGTAACGAT

Linker_R /5phos/ACC ATCGTTACGTCGG

6) After ligation, amplify each small amplicon with the following primers. Underlined are illumina sequencing primer region.

Amplicon	Forward Primer	Reverse Primer
Amplicon 1	GCTGAACCGCTCTTCCGATCT **TGCTCAAGGMAMCTCTATGT (534-553)**	AATGATACGGCGACCACCGAGATCT ACACTCTTTCCCTACACGAC
Amplicon 2	GCTGAACCGCTCTTCCGATCT **TTTMGRAAACTKCCTGTWAAT (945-965)**	AATGATACGGCGACCACCGAGATCT ACACTCTTTCCCTACACGAC

95 ℃ 30 sec, 56℃ 30sec, 68℃ 40sec 20 cycles

7) Final amplification with the sequencing adaptor:

forward	CAAGCAGAAGACGGCATACGAGATCGGTCTCGGCATTCCTGCTGAACC
reverse	AATGATACGGCGACCACCGAGATCTACACTCTTTCCCTACACGAC

95 ℃ 30 sec, 56℃ 30sec, 68℃ 40sec 15 cycles

8) Sequencing the amplified product with Illumina PE250.
